# Determinants of Early Introduction of Solid, Semi-Solid or Soft Foods among Infants Aged 3–5 Months in Four Anglophone West African Countries

**DOI:** 10.3390/nu6072602

**Published:** 2014-07-14

**Authors:** Abukari I. Issaka, Kingsley E. Agho, Andrew N. Page, Penelope Burns, Garry J. Stevens, Michael J. Dibley

**Affiliations:** 1 School of Science and Health, University of Western Sydney, Penrith NSW 2751, Australia; E-Mails: k.agho@uws.edu.au (K.E.A.); a.page@uws.edu.au (A.N.P.); 2 School of Medicine, University of Western Sydney, Penrith NSW 2751, Australia; E-Mails: p.burns@uws.edu.au (P.B.); G.Stevens@uws.edu.au (G.J.S.); 3 Sydney School of Public Health, Edward Ford Building (A27), University of Sydney, NSW 2006, Australia; E-Mail: michael.dibley@sydney.edu.au

**Keywords:** semi-solid foods, malnutrition, breastfeeding, Anglophone, West Africa

## Abstract

This study was conducted to explore and identify factors associated with the practice of early introduction of solid, semi-solid or soft foods among infants aged 3–5 months in four Anglophone West African countries. Data sources for the analyses were the latest Demographic and Health Survey datasets of the 4 countries, namely Ghana (GDHS, 2008), Liberia (LDHS, 2007), Nigeria (NDHS, 2013) and Sierra Leone (SLDHS, 2008). Multiple logistic regression methods were used to analyze the factors associated with early introduction of solid, semi-solid or soft foods among infants aged 3–5 months, using individual-, household- and community-level determinants. The sample consisted of 2447 infants aged 3–5 months from four Anglophone West African countries: 166 in Ghana, 263 in Liberia, 1658 in Nigeria and 360 in Sierra Leone. Multivariable analyses revealed the individual factors associated with early introduction of solid, semi-solid or soft foods in these countries. These included increased infant’s age, diarrhea, acute respiratory infection and newborns perceived to be small by their mothers. Other predictors of early introduction of solid, semi-solid or soft foods were: mothers with no schooling, young mothers and fathers who worked in an agricultural industry. Public health interventions to improve exclusive breastfeeding practices by discouraging early introduction of solid, semi-solid or soft foods are needed in all 4 countries, targeting especially mothers at risk of introducing solid foods to their infants early.

## 1. Introduction

It is an established fact that breastfeeding is crucial for sustaining the health and well-being of newborns and infants. Infants who are appropriately breastfed grow better and are often less likely to fall sick or die, compared to those who are introduced to complementary feeding early [1,2,3,4]. However, in most countries, only a relatively small percentage of mothers practice optimal breastfeeding behaviors due to cultural and religious practices. Optimal breastfeeding entails breastfeeding in the first hour after birth and exclusive breastfeeding (EBF) for the first 6 months of life. However, after 6 months of an infant’s life, breast milk alone is nutritionally insufficient [5], and complementary feeding (practice of offering an infant solid, semi-solid or soft foods) has to be introduced at this juncture, alongside breastfeeding for at least 2 years. This is to ensure the achievement of the infant’s optimal growth, development and health [6,7].

Timeliness of introduction of solid, semi-solid or soft foods is very important to the health and well-being of the child. According to the new World Health Organization (WHO) indicators, the timeliness is assessed by whether infants aged 6–8 months receive solid, semi-solid or soft foods, irrespective of being breastfed or not [8,9]. Due to the fact that most mothers do not exclusively breastfeed their infants, it can be inferred that complementary feeding commences earlier than recommended in a sizable number of infants, in both developed and developing countries [9,10]. Early introduction of solid, semi-solid or soft foods (EISF) tends to cause a displacement of breast milk and does not confer any growth advantage over EBF [11]. The extant literature [12,13,14,15] is replete with evidence that reveal that EISF was associated with diarrhea and respiratory infections in infants.

According to Demographic and Health Surveys (DHS) reports of the 4 countries under study [16,17,18,19], the percentage of infants aged under 6 months that were exclusively breastfed was small (63%, 34%, 13.1% and 11.2% for Ghana, Liberia, Nigeria and Sierra Leone respectively). This suggested that most mothers practiced EISF in these countries, especially in Liberia, Nigeria and Sierra Leone. The low EBF rates in these countries is reflected in the number of under-5 deaths (thousands) [20]: Ghana, 2012 (56), Liberia, 2012 (11), Nigeria, 2012 (827) and Sierra Leone, 2012 (39). Addressing the problem of early introduction of solid, semi-solid or soft foods in these countries could go a long way towards preventing or reducing the burden of infant morbidity and mortality, and therefore promote the achievement of the fourth Millennium development Goal (MGD) of child survival.

The aim of this study was to examine the prevalence of EISF to infants aged 3–5 months, and to determine individual-, household- and community-level characteristics associated with EISF among these infants across the 4 Anglophone West African countries, using the most recent DHS data of the countries.

## 2. Methods

The most recent DHS datasets of the four countries under examination were used in all the analyses in this study [16,17,18,19]. The various DHS are nationally representative household surveys which adopt a multistage stratified cluster sampling design. Analyses in this study were limited to infants aged between 3 and 5 months who lived with the respondent (ever-married women aged 15–49 years). The total weighted sample sizes for the various countries were: 166 (Ghana), 263 (Liberia), 1599 (Nigeria) and 360 (Sierra Leone). The average response rate for the surveys was 95.6%. Details of survey methodology, sampling procedure and questionnaire can be found in the respective DHS reports.

### 2.1. Outcome Indicator and Explanatory Variables

The outcome variable for this study was EISF, which was defined as the proportion of infants younger than 6 months of age who received solid, semi-solid or soft foods during the past 24 h. This outcome variable was examined against a set of independent variables, which were classified into 3 levels, namely, individual, household and community. The individual-level attributes of the infant included their sex, age (in completed months) and size at birth as perceived by the mother. The individual-level characteristics of the mother included her age (in years), work status, highest level of education achieved, religion, marital status and access to the media. Other individual-level characteristics involved the mother-infant dyad: number of antenatal clinic visits, place of delivery, the mode of delivery, birth order and timing of postnatal contacts with health-care providers.

Household-level characteristics comprised wealth index of the household and the source of drinking water. The household wealth index was constructed using a method recommended by the World Bank Poverty Network and United Nations Children’s Fund [21]. The index was divided into five quintiles, namely: poorest, poorer, middle, richer and richest. In this study, this index was divided into three categories. The bottom 40% of households (poorest and poorer) was arbitrarily referred to as poor households (poor), the next 40% as the middle households (middle) and the top 20% of households (richer and richest) as rich households (rich).

The type of residence (urban or rural) and administrative or geographical region constituted the community-level characteristics.

### 2.2. Statistical Analyses

Early introduction of solid, semi-solid or soft foods was expressed as a dichotomous outcome; early introduction of solid, semi-solid or soft foods was expressed as category 1 and category 0 for timely introduction complementary foods for infants aged 6–8 months. Stata version 12.0 (Stata Corp., College Station, TX, USA) was used to perform statistical analyses in this study. The “svy” (survey) tabulation was applied to allow for adjustments for the cluster sampling design, sampling weights and the calculation of standard errors. The Taylor series linearization method was used in the surveys when estimating confidence intervals around prevalence estimates and the significance of associations was tested with a χ^2^ test. simple and multiple logistic regression methods were used in a stepwise backward regression model to determine the factors that were significantly associated with early introduction of solid, semi-solid or soft foods. A stepwise backward elimination approach was applied, which was validated by using the following procedures: (1) the main risk factors with *p*-value < 0.20 were initially entered in the backward elimination process, (2) the backward elimination was tested by including all variables (all potential risk factors); and, (3) any collinearity between study factors were tested and reported in the final model. In order to assess the adjusted risk of independent variables, the odds ratios with 95% confidence intervals were calculated. The variables with a *p*-value less than 0.05 were retained in the final model. Separate logistic regression models were run for each country.

## 3. Results

### 3.1. Characteristics of the Samples

As shown in Table 1, the majority of mothers in Liberia and Sierra Leone had no schooling. Less than one half of the mothers in all four countries attained secondary education or higher. With the exception of Liberia, more mothers from the other countries reported their babies to be large at birth. In all the countries, the majority of mothers were in paid employment in the past 12 months, were currently married, were aged between 25 and 34 years, delivered their babies through non-caesarean section, were in their 20s at the time of delivery, were illiterate, did not read newspapers/magazines, and belonged to poor households and resided in rural areas. With the exception of mothers in Ghana, majority in the other countries delivered their babies at home. The majority of mothers in Liberia and Sierra Leone delivered their babies with the help of traditional birth attendants (TBAs).

**Table 1:** Individual-, household- and community-level characteristics of infants aged 3–5 months in 4 Anglophone West African countries, 2007–2013.

Characteristic	Ghana	Liberia	Nigeria	Sierra Leone
	*n* = 166	*n* = 263	*n* = 1658	*n* = 360
*Individual-level factors*				
Mother’s work status				
Non-working	32.8	34.3	42.3	39.7
Working (past 12 months)	67.3	65.7	57.7	60.3
Father’s occupation				
Non Agricultural	48.4	30.0	59.2	87.6
Agricultural	40.9	50.3	36.1	
Not working	10.6	19.8	4.7	12.4
Mother’s education				
No education	27.4	52.0	44.8	75.7
Primary	28.3	34.4	22.3	11.3
Secondary and higher	44.4	13.6	32.9	13.0
Father’s education				
No education	27.5	27.2	36.4	71.6
Primary	8.5	26.1	21.4	10.7
Secondary and higher	64.0	46.7	42.3	17.7
Perceived size of baby at birth				
Small	9.1	20.4	14.8	22.1
Average	23.8	42.4	38.0	30.1
Large	67.0	37.2	47.2	47.9
Mother’s age (years)				
15–24	35.2	35.3	34.6	27.8
25–34	40.8	47.0	47.0	47.7
35–49	24.0	17.8	18.4	24.5
Mother’s marital status				
Currently married	84.5	79.5	95.5	87.0
Formerly married *	15.5	20.5	4.5	13.0
Mother’s religion				
Christian	70.5	87.8	43.1	19.8
Muslim and others	29.5	12.2	56.9	80.2
Birth order of infant				
First-born	26.7	13.5	21.2	19.7
2nd–4th	46.2	53.8	44.0	47.9
5 or more	27.2	32.8	34.7	32.4
Preceding birth interval				
No previous birth	26.7	13.5	21.3	19.7
<24 months	5.3	7.9	14.1	9.6
>24 months	68.1	78.6	64.6	70.8
Baby’s sex				
Male	53.8	56.4	49.6	51.2
Female	46.2	43.6	50.4	48.8
Infant’s age (months)				
Mode of delivery				
Non-caesarean	94.3	93.3	98.4	98.5
Caesarean	5.7	6.7	1.6	1.5
Mother’s BMI (kg/m^2^)				
≤18	3.4	4.1	7.3	11.2
18–25	63.5	80.1	72.8	66.8
>25	33.2	15.7	19.9	22.0
Mother’s age at infant’s birth				
<20	17.1	10.6	14.2	15.2
20–29	43.8	47.3	53.6	46.5
30–39	33.1	38.5	26.3	34.2
>40	6.1	3.7	5.9	4.1
Place of delivery				
Home	42.9	58.1	62.8	76.2
Health facility	57.1	41.9	37.2	23.8
Infant had diarrhea (in past 2 weeks)				
No	85.5	86.7	89.8	89.2
Yes	14.5	13.3	10.2	10.8
Infant had ARI (in past 2 weeks)				
No	9.8	87.3	95.0	85.9
Yes	8.0	12.7	5.0	14.1
Infant had fever (in the past 2 weeks)				
No	90.4	75.1	87.6	74.2
Yes	9.6	24.9	12.4	25.8
Type of delivery assistance				
Health professional	61.2	43.3	50.3	38.4
Traditional birth attendant	33.1	47.5	26.6	46.4
Other untrained personnel	5.7	9.2	23.1	15.2
Antenatal Clinic visits				
None	2.7	4.2	39.1	10.9
1–3	23.6	21.9	13.0	29.8
4+	73.7	73.9	47.9	59.3
Timing of postnatal check-up				
No check-ups (including missing)	20.5	31.1	56.0	38.0
0–2 days	43.9	43.9	26.0	34.2
3–6 days	11.7	6.7	7.6	9.2
7+ days	23.9	18.4	10.4	18.6
Maternal literacy				
No	66.0	69.5	58.5	85.5
Yes	34.0	30.5	41.5	14.5
Mother read newspaper/magazine				
No	86.7	84.1	84.2	94.9
Yes	13.3	15.9	15.8	5.1
Mother listened to the radio				
No	24.0	48.6	36.3	58.7
Yes	76.1	51.4	63.7	41.3
Mother watched television				
No	38.5	70.0	58.3	89.4
Yes	61.5	30.0	41.7	10.6
*Household-level factors*				
Household Wealth Index				
Poor	41.8	49.6	45.5	48.8
Middle	16.8	14.9	18.8	21.2
Rich	41.4	35.5	35.7	30.0
Source of drinking water				
Unprotected	19.5	36.6	52.7	56.7
Protected	80.5	63.5	47.3	43.3
*Community-level factors*				
Residence				
Urban	40.0	28.9	30.8	24.2
Rural	60.0	71.1	69.2	75.8
Geographical/Administrative region				
1	7.7	19.0	12.8	20.6
2	12.8	11.2	17.5	50.4
3	15.1	18.3	29.5	16.2
4	8.5	7.9	8.9	12.9
5	11.1	8.0	14.1	
6	12.7	35.7	17.2	
7	8.4			
8	16.6			
9	5.6			
10	1.6			
1 = Western (Ghana), Monrovia (Liberia), North Central (Nigeria) and Eastern (Sierra Leone)				
2 = Central (Ghana), North Western (Liberia), North East (Nigeria) and Northern (Sierra Leone)				
3 = Greater Accra (Ghana), South Central (Liberia), North West (Nigeria) and Southern (Sierra Leone)				
4 = Volta (Ghana), South Eastern (Liberia), South East (Nigeria) and Western (Sierra Leone)				
5 = Eastern (Ghana), South Eastern (Liberia) and South West (Nigeria)				
6 = Ashanti (Ghana), North central (Liberia) and South South (Nigeria)				
7 = Brong Ahafo (Ghana)				
8 = Northern (Ghana)				
9 = Upper East (Ghana)				
10 = Upper East (Ghana)				

* Included divorced, separated or widowed.

### 3.2. Early Introduction of Solid, Semi-Solid or Soft Foods

As shown in Table 2, EISF rates were not significantly different between working and nonworking mothers in all the countries except in Nigeria where mothers in paid employment reported a significantly higher rate. A majority of mothers who had secondary education or higher reported higher EISF rates in all the countries except in Liberia, although these differences were not significant. In Liberia and Nigeria, EISF rates were significantly higher among mothers who had between 1 and 3 antenatal clinic visits. The EISF rate was significantly higher among infants in Liberia whose fathers were unemployed. In Nigeria, EISF rates were significantly higher among infants whose mothers did not listen to the radio but watched television. EISF rate was significantly higher among babies in Sierra Leone whose mothers perceived them to be small at birth.

Within Liberia and Nigeria, significant differences were observed across geographical regions. For instance, the South West region in Nigeria reported significantly higher EISF rate (57.7%) than the other 5 regions. In Liberia, the Monrovia region reported the highest EISF rate (51.3%) whereas the North Western region had the lowest (4.3%).

**Table 2:** Early introduction of solid, semi-solid or soft foods among infants aged 3–5 months by Individual-, Household- and Community-level characteristics in 4 Francophone West African countries, 2007–2013.

Characteristic	Ghana	Liberia	Nigeria	Sierra Leone
	% [95% CI]	% [95% CI]	% [95% CI]	% [95% CI]
*Individual-level factors*				
Mother’s work status				
Non-working	15.5 [7.9, 8.2]	22.5 [13.1, 35.8]	32.8 [28.5, 37.4]	37.6 [28.0, 48.2]
Working (past 12 months)	28.2 [19.7, 38.6]	35.5 [26.0, 46.2]	32.4 [28.5, 36.6]	38.4 [31.4, 46.0]
Father’s occupation				
Non Agricultural	22.7 [13.8, 35.1]	21.5 [11.4, 36.8]	32.0 [28.8, 35.3] ^§^	37.4 [31.6, 43.5]
Agricultural	26.3 [16.3, 39.6]	31.8 [22.7, 42.4]		
Not working	21.4 [7.5, 47.6]	43.6 [28.5, 60.0]	49.0 [33.3, 64.8]	42.1 [25.9, 60.3]
Mother’s education				
No education	19.1 [9.1, 35.9]	29.3 [20.3, 40.3]	35.2 [30.5, 40.1]	36.0 [29.7, 43.0]
Primary	29.5 [17.1, 45.8]	31.3 [20.7, 44.4]	32.1 [26.3, 38.6]	35.1 [19.8, 54.3]
Secondary and higher	23.6 [15.0, 35.1]	36.5 [18.4, 59.4]	28.8 [24.6, 33.4]	51.5 [35.6, 67.2]
Father’s education				
No education	19.3 [10.0, 33.9]	13.5 [6.4, 26.1] §	37.8 [32.7, 43.3] ^§^	35.2 [28.6, 42.4]
Primary	29.4 [7.6, 67.7]	42.0 [25.3, 60.8]	32.0 [25.0, 39.8]	35.8 [19.3, 56.5]
Secondary and higher	24.3 [15.8, 35.4]	30.6 [20.5, 43.0]	26.3 [22.4, 30.7]	43.6 [30.7, 57.5]
Perceived size of baby at birth				
Small	16.2 [5.0, 41.5]	41.2 [26.3, 57.8]	28.7 [22.5, 35.8]	45.9 [33.6, 58.8] ^§^
Average	21.1 [10.7, 37.4]	34.0 [21.8, 48.7]	33.0 [28.7, 37.7]	25.8 [17.7, 36.0]
Large	26.3 [18.3, 36.3]	22.5 [14.8, 32.8]	33.3 [28.7, 38.2]	43.2 [35.1, 51.7]
Mother’s age (years)				
15–24	28.4 [17.7, 42.3]	32.8 [20.6, 47.9]	34.7 [29.6, 40.2]	43.9 [32.2, 56.4]
25–34	22.6 [12.7, 37.0]	31.0 [21.7, 42.0]	30.8 [26.6, 35.4]	36.9 [28.8, 45.8]
35–49	19.9 [10.1, 35.5]	27.6 [15.8, 43.5]	33.3 [26.7, 40.7]	33.2 [24.0, 44.0]
Marital status				
Currently married	24.0 [16.7, 33.2]	28.3 [20.9, 37.1]	31.8 [28.7, 35.2] §	37.4 [31.6, 43.5]
Formerly married *	24.3 [10.5, 46.7]	41.6 [27.4, 57.3]	53.7 [38.4, 68.3]	41.8 [25.6, 60.0]
Mother’s religion				
Christian	26.7 [18.1, 37.4]	30.9 [23.1, 40.0]	35.6 [30.9, 40.5]	44.4 [31.9, 57.6]
Muslim and others	17.7 [10.0, 29.4]	31.8 [15.8, 53.8]	30.9 [26.8, 35.3]	36.4 [29.8, 43.4]
Birth order of infant				
First-born	23.8 [12.6, 40.4]	34.8 [18.2, 56.2]	30.1 [24.1, 36.9]	36.2 [23.6, 50.9]
2nd–4th	26.4 [16.5, 39.5]	30.6 [21.0, 42.2]	32.1 [27.9, 36.7]	40.1 [31.8, 49.0]
5 or more	20.2 [11.1, 33.9]	30.1 [20.6, 41.7]	34.4 [29.65, 39.5]	35.9 [26.3, 46.8]
Preceding birth interval				
No previous birth	23.8 [12.6, 40.4]	34.8 [18.2, 56.2]	30.1 [24.1, 36.9]	36.2 [23.6, 50.9]
<24 months	40.5 [15.8, 71.2]	40.0 [18.3, 66.6]	32.2 [24.9, 40.4]	53.7 [34.5, 71.8]
>24 months	22.8 [15.3, 32.6]	29.4 [22.0, 38.2]	33.2 [29.6, 37.1]	36.3 [29.9, 43.3]
Infant’s sex				
Male	21.6 [13.2, 33.5]	29.5 [20.5, 40.4]	34.2 [30.4, 38.3]	35.9 [28.6, 43.8]
Female	26.8 [17.5, 38.8]	33.0 [23.4, 44.2]	30.8 [26.6, 35.3]	40.2 [31.7, 49.2]
Infant’s age (months)				
Mode of delivery				
Non-caesarean	24.4 [17.4, 33.1]	30.0 [22.4, 38.9]	32.3 [29.2, 35.7]	38.4 [32.6, 44.5]
Caesarean	18.3 [2.7, 64.3]	44.9 [19.3, 73.5]	43.4 [26.3, 62.3]	12.2 [1.6, 54.8]
Mother’s BMI (kg/m^2^)				
≤18	0	47.0 [16.1, 80.4]	35.4 [25.2, 47.2]	31.3 [12.1, 60.1]
18–25	24.9 [16.6, 35.6]	29.8 [22.3, 38.4]	32.9 [29.2, 36.8]	40.4 [29.5, 52.4]
>25	25.8 [14.4, 41.8]	34.3 [20.3, 51.7]	30.1 [24.7, 36.2]	18.2 [8.2, 35.7]
Mother’s age at infant’s birth				
<20	19.9 [8.7,39.2]	23.5 [8.1,51.9]	35.3 [27.6,43.8]	54.0 [37.2,70.1]
20–29	30.6 [19.8,44.0]	33.3 [22.8,45.9]	31.7 [27.6,36.2]	33.8 [26.3,42.3]
30–39	17.1 [9.1,29.9]	29.9 [20.1,42.0]	31.8 [27.1,36.8]	36.7 [27.5,46.9]
>40	26.0 [6.6,63.6]	34.1 [10.3,70.1]	37.7 [27.0,49.9]	36.3 [15.3,64.2]
Place of delivery				
Home	25.3 [15.9, 37.6]	33.9 [24.1, 45.3]	34.6 [30.6, 38.9]	38.7 [31.9, 46.0]
Health facility	23.1 [14.7, 34.4]	27.1 [17.3, 39.7]	28.8 [24.4, 33.7]	35.5 [25.4, 47.0]
Infant had diarrhea (in past 2 weeks)				
No	24.7 [17.4, 33.8]	26.2 [18.8, 35.3] ^§^	31.9 [28.7, 35.4]	37.7 [31.5, 44.3]
Yes	20.0 [7.4, 43.9]	62.1 [44.0, 78.8]	39.9 [30.4, 50.3]	40.0 [25.2, 57.0]
Infant had ARI (in past 2 weeks)				
No	24.1 [9.1, 50.1]	33.0 [24.9, 42.2]	32.5 [29.4, 35.8]	37.6 [31.5, 44.1]
Yes	26.0 [8.1, 58.3]	17.6 [7.7, 35.3]	34.2 [21.2, 50.1]	40.1 [26.0, 56.1]
Infant had fever (in the past 2 weeks)				
No	23.1 [16.4, 31.6]	28.5 [20.6, 37.8]	32.5 [29.3, 35.8]	35.1 [28.4, 42.5]
Yes	32.5 [14.0, 58.8]	38.7 [25.5, 53.8]	33.3 [23.9, 44.2]	46.1 [35.0, 57.7]
Type of delivery assistance				
Health professional	22.6 [14.4, 33.6]	25.9 [16.7, 37.8]	32.1 [28.4, 36.1] §	35.6 [27.4, 44.8]
Traditional birth attendant	28.5 [17.5, 42.9]	34.0 [22.4, 47.8]	37.8 [31.5, 44.6]	40.3 [31.6, 49.6]
Other untrained personnel	26.2 [6.3, 65.4]	29.8 [12.5, 55.7]		34.9 [18.7, 55.6]
Antenatal Clinic visits				
None	8.7 [1.0, 46.6]	26.9 [10.1, 54.5] ^§^	36.4 [31.1, 42.2]	40.2 [22.6, 60.8]
1–3	23.8 [12.7, 40.2]	52.0 [37.3, 66.3]	32.8 [26.2, 40.1]	45.8 [32.7, 59.5]
4+	24.7 [16.4, 35.3]	22.0 [15.5, 30.4]	30.6 [26.7, 34.8]	35.3 [27.3, 44.2]
Timing of postnatal check-up				
No check-ups (including missing)	18.5 [8.4, 36.0]	24.7 [15.3, 37.3]	34.7 [30.8, 38.9]	37.6 [27.0, 49.6]
0–2 days	30.1 [19.1, 43.9]	34.4 [24.0, 46.6]	28.7 [23.9, 34.1]	39.8 [31.0, 49.3]
3–6 days	20.9 [8.3, 43.6]	20.6 [5.4, 54.0]	30.5 [20.0, 43.7]	32.1 [17.5, 51.3]
7+ days	19.2 [9.3, 35.6]	37.4 [20.4, 58.2]	29.7 [20.2, 41.4]	38.1 [24.6, 53.7]
Maternal literacy				
No	25.5 [17.2, 36.2]	28.9 [20.5, 39.1]	36.0 [31.8, 40.5] ^§^	36.1 [30.2, 42.4]
Yes	21.1 [11.9, 34.7]	35.8 [22.4, 52.0]	27.2 [23.5, 31.3]	49.0 [33.4, 64.9]
Mother read newspaper/magazine				
No	23.6 [16.5, 32.6]	29.7 [21.9, 38.9]	33.4 [30.0, 37.1]	37.0 [31.2, 43.3]
Yes	26.8 [11.6, 50.6]	38.1 [18.5, 62.5]	27.1 [20.9, 34.2]	55.0 [31.0, 76.9]
Mother listened to the radio				
No	15.8 [7.2, 31.4]	30.4 [20.9, 41.8]	36.5 [31.4, 41.9] ^§^	38.0 [30.3, 46.5]
Yes	26.6 [18.5, 36.7]	31.6 [22.3, 42.7]	30.0 [26.6, 33.7]	37.8 [29.4, 47.0]
Mother watched television				
No	20.7 [12.4, 32.5]	32.1 [23.3, 42.3]	36.1 [31.9, 40.5] ^§^	37.4 [31.3, 43.9]
Yes	26.1 [17.4, 37.2]	28.5 [16.9, 43.8]	28.0 [24.1, 32.3]	42.9 [28.6, 58.5]
*Household-level factors*				
Household Wealth Index				
Poor	22.5 [13.0, 35.9]	30.4 [20.2, 43.1]	36.5 [28.8, 44.9]	36.5 [28.8, 44.9]
Middle	24.3 [15.4, 36.3]	27.5 [18.3, 39.2]	36.4 [27.1, 46.8]	36.4 [27.1, 46.8]
Rich	27.4 [11.2, 53.1]	44.1 [22.5, 68.2]	50.7 [35.4, 65.8]	50.7 [35.4, 65.8]
Source of drinking water				
Unprotected				
Protected				
*Community-level factors*				
Residence				
Urban	23.4 [13.7, 37.0]	36.3 [23.5, 51.4]	26.6 [21.8, 32.1] ^§^	42.7 [31.1, 55.1]
Rural	24.5 [15.9, 35.7]	28.9 [20.4, 39.1]	35.8 [32.0, 39.8]	36.4 [30.0, 43.4]
Geographical/Administrative region				
1	24.2 [6.7, 58.7]	51.3 [33.4, 68.8] ^§^	41.5 [32.8, 50.7] ^§^	30.6 [21.0, 42.2]
2	29.3 [13.7, 52.1]	4.3 [1.1, 15.7]	28.4 [20.8, 37.5]	37.6 [29.1, 46.9]
3	27.2 [10.3, 55.0]	37.1 [16.6, 63.7]	30.9 [26.3, 36.0]	37.7 [25.3, 52.1]
4	20.7 [5.1, 55.7]	28.7 [12.4, 53.2]	38.8 [27.9, 50.9]	51.4 [35.0, 67.6]
5	20.1 [7.2, 44.7]	18.5 [10.2, 31.2]	42.4 [34.1, 51.1]	
6	39.4 [19.0, 64.3]	28.8 [19.3, 40.7]	21.2 [15.2, 28.7]	
7	29.6 [5.6, 74.8]			
8	11.7 [4.3, 28.2]			
9	8.3 [1.1, 42.3]			
10	28.1 [9.2, 60.2]			
1 = Western (Ghana), Monrovia (Liberia), North Central (Nigeria) and Eastern (Sierra Leone)				
2 = Central (Ghana), North Western (Liberia), North East (Nigeria) and Northern (Sierra Leone)				
3 = Greater Accra (Ghana), South Central (Liberia), North West (Nigeria) and Southern (Sierra Leone)				
4 = Volta (Ghana), South Eastern (Liberia), South East (Nigeria) and Western (Sierra Leone)				
5 = Eastern (Ghana), South Eastern (Liberia) and South West (Nigeria)				
6 = Ashanti (Ghana), North central (Liberia) and South South (Nigeria)				
7 = Brong Ahafo (Ghana)				
8 = Northern (Ghana)				
9 = Upper East (Ghana)				
10 = Upper East (Ghana)				

* Included divorced, separated or widowed, ^§^ statistically significant.

### 3.3. Factors Associated with Early Introduction of Solid, Semi-Solid or Soft Foods According to Multivariate Modeling

Table 3 shows the adjusted odds ratios for factors associated with EISF based on multivariate modeling statistical analyses. As expected, increasing infant age was consistently associated with significantly high EISF rates in all the countries except in Liberia, and the odds ratios ranged from 1.44 in Nigeria to 2.39 in Sierra Leone. Infants in Liberia who contracted diarrhea in the past 24 h and whose fathers worked in an agricultural sector had significantly higher odds for EISF. In Nigeria, 5th or higher-order born infants had significantly higher odds for EISF, whilst in Sierra Leone, infants who were delivered through non-caesarean section, and whose mothers were aged 20 years or less had significantly higher odds for EISF. The odds for EISF were significantly higher among infants in Nigeria whose mothers had no schooling; and in Liberia, infants whose fathers had secondary education or higher were predictors of early introduction of solid, semi-solid or soft foods. Infants in Liberia and Sierra Leone whose mothers perceived them to be small at birth were significantly more likely to experience EISF.

**Table 3:** Factors associated with early introduction of solid, semi-solid or soft foods among infants aged 3–5 months in four Anglophone West African countries, 2007–2013.

Country	Variable	OR	[95% CI]	*p*
Ghana	Infant’s age (in months)	1.87	[1.12, 3.12]	0.017
Liberia	Infant had diarrhea (in past 2 weeks)			
	No	1.00		
	Yes	8.56	[3.02, 24.32]	<0.001
	Administrative region	See details below ^a^		
	Father’s occupation			
	Non Agricultural	1.00		
	Agricultural	5.06	[1.29, 19.79]	0.020
	Not working	2.02	[0.33, 12.23]	0.441
	Father’s education			
	No education	1.00		
	Primary	3.40	[1.19, 9.75]	0.023
	Secondary and higher	5.01	[1.87, 13.46]	0.002
	Perceived size of baby at birth			
	Small	1.00		
	Average	0.28	[0.11, 0.70]	0.006
	Large	0.18	[0.07, 0.49]	0.001
Nigeria	Geographical region	See details below ^b^		
	Mother’s education			
	No education	1.00		
	Primary	0.92	[0.66, 1.28]	0.606
	Secondary and higher	0.66	[0.46, 0.95]	0.027
	Birth order of infant			
	First-born	1.00		
	2nd–4th	1.05	[0.79, 1.39]	0.744
	5 or more	1.37	[1.01, 1.86]	0.046
	Infant’s age (in months)	1.44	[1.24, 1.68]	<0.001
Sierra Leone	Mode of delivery			
	Non-caesarean	1.00		
	Caesarean	0.11	[0.01, 0.94]	0.044
	Geographical region	See details below ^c^		
	Perceived size of baby at birth			
	Small	1.00		
	Average	0.41	[0.19, 0.88]	0.021
	Large	0.86	[0.49, 1.52]	0.602
	Mother’s age at infant’s birth (years)			
	<20	1.00		
	20–29	0.37	[0.14, 0.96]	0.041
	30–39	0.48	[0.19, 1.26]	0.136
	>40	0.33	[0.08, 1.40]	0.131
	Infant’s age (in months)	2.39	[1.61, 3.54]	<0.001

^a^ Compared to the Monrovia region, the odds of early introduction of solid, semi-solid or soft foods was significantly lower in the North Western (OR = 0.03, 95% CI: [0.00, 0.18]), South Eastern b (OR = 0.15, 95% CI: [0.02, 0.90]) and North Central (OR = 0.14, 95% CI: [0.03, 0.73]) regions in Liberia; ^b^ Compared to the North Central region, the odds of early introduction of solid, semi-solid or soft foods was significantly lower in the North West (OR = 0.39, 95% CI: [0.26, 0.58]) and the South West (OR = 1.83, 95% CI: [1.11, 3.00]) regions in Nigeria; ^c^ Compared to the Eastern region, the odds of early introduction of solid, semi-solid or soft foods was significantly higher in the Western (OR = 4.09, 95% CI: [1.66, 10.10]) region in Sierra Leone. CI: Confidence interval. *p*: *p* value.

Infants whose mothers resided in the Monrovia region in Liberia, the South West region in Nigeria and the Western region in Sierra Leone had significantly higher odds for EISF.

## 4. Discussion

In this study, factors which were associated with EISF included age of the infant, illness, parents’ level of education, mother’s age at the time of giving birth, perceived size of the baby, geographical region, birth order of the infant as well as the mode of delivery of the baby. It was found that children in Liberia who contracted diarrhea in the past 24 h were significantly more likely to receive EISF. Increasing age of the infant was found to be significantly associated with EISF in almost all 4 countries. In Nigeria, EISF rates were found to be significantly higher among infants whose mothers had no schooling. In Liberia, infants whose fathers worked in an agricultural industry were predictors of EISF.

Since a high rate of EISF implies low EBF rates, identifying the factors that raise EISF rates could be used to inform governments, policy makers and other stakeholders in the various countries to draft future intervention programs for improving EBF practices among mothers in these West African countries.

One of the main strengths of this study is that it used the most recent DHS datasets of the various countries, which are nationally representative surveys that use standardized methods to achieve high response rates. The main limitations were that the sample sizes were small, which could have resulted in a limited number of variables being statistically associated with the practice of EISF, and the cross-sectional approach used in this study, which could limit the ability to draw inferential causalities from the predictor variables and the practice of EISF in these countries [22]. Another limitation of this study was that the EISF was based on a 24-hour recall rather than the situation over the entire period from birth. In that case, infants who were given pre-lacteals or other foods earlier than the 24 h could have also been classified as being introduced to solid, semi-solid or soft foods early, thus, underestimating the real situation. A prospective study design where exposures were measured prior to outcomes would be an alternative.

In our study, we found that EISF rates were significantly higher among infants in Liberia, who contracted diarrhea the previous day. This finding is consistent with a separate finding in Vietnam [23] where contraction of diarrhea by an infant was found to be a predictor of EISF.

Previous studies [24,25] have shown that there was a high risk of non-EBF among working mothers, which may imply that the risk of higher rates of EISF was high among working mothers. No association was found between the working status of mothers and EISF. Our study found that EISF rates were significantly high among infants in Sierra Leone, whose mothers were aged 20 years or younger at the time of giving birth. This is consistent with earlier studies where younger mothers [26,27,28,29,30] were found to be significantly most likely to introduce solid, semi-solid or soft foods early. Obviously, such mothers lacked the required experience to practice appropriate infant feeding. This finding calls for more attention to be paid to first time mothers, who are less experienced with regards to appropriate practices of breastfeeding of infants. It is suggested that breastfeeding support groups should be formed at community or village levels in all four countries to offer practical solutions to the inappropriate infant feeding practices. The formation of such mother support groups has occurred in Cambodia [31], where village leaders, traditional birth attendants, experienced mothers and health professionals participate at the community level, and this has contributed to relatively low EISF rates due to relatively higher EBF rates in that country. Consistent with earlier studies [27,29,30], we found a significant association between EISF and maternal level of education. Mothers in Nigeria who had no schooling were found to be a significant predictor of EISF. A cohort study [32] on predictors of early introduction of solid foods found that women with less than 12 years of education were significantly more likely to introduce solids before 17 weeks than those with more than 12 years of education. This emphasizes the role education plays in appropriate infant feeding practices. This study thus identified less educated mothers or mothers with no schooling as a potential target group for future education and support programs.

In the current study, EISF rates were found to significantly increase with increasing age of the infant in 3 of the 4 countries examined. This implied that there was a drop in EBF rates with age of the infant in these countries, which is consistent with findings from a separate study in 5 East and Southeast Asian countries [24]. Appropriate measures should therefore be taken to discourage mothers from introducing solid, semi-solid or soft foods too early.

There is evidence in the literature [33], which showed that smaller newborns were more likely to receive infant formula and other infusions to stabilize their condition. This is consistent with a finding in this study, where the risk of EISF was found to be significantly higher among infants in Liberia and Sierra Leone who were perceived to be small by their mothers.

Evidence in the literature [34,35] found no relationship between the age of introduction of solids and birth order of the infant. However, in this study, we found a significant association between birth order and early introduction of solids. In Nigeria, the risk of EISF was found to be significantly higher among 5th or higher born infants compared to 1st, 2nd, 3rd or 4th born infants. This finding may reflect complacency on the part of the mother, who may reckon she would have been experienced enough to try to introduce the infant to solid foods early.

A recent study in Ghana [22] found that there was a regional difference in the rates of EBF (and therefore EISF). Regional differences in the rates of EISF have been reported in Pakistan [36]. Although this finding was not revealed for Ghana in this study, rates of EISF were found to vary in the geographical regions in Liberia, Nigeria and Senegal. These differences may be attributed to different cultural beliefs, differences in access to information regarding infant feeding practices or simply myths. There is the need for further studies to explore the effects of these factors on infant feeding practices in the different regions of the countries concerned.

## 5. Conclusions

Results of this study identified the main factors that were significantly associated with early introduction of solid, semi-solid or soft foods to be: contraction of diarrhea by the infant, babies perceived to be small at birth, a mother with no schooling, increasing age of the infant, mothers who gave birth when they were aged 20 years or younger and geographical regional differences within country. Although in West Africa children are breastfed for a long time as reflected by the fact that the mean duration of breastfeeding in West and Central African countries is 20 months (in 2007), the exclusive breastfeeding rate was lower than in any other region in the world [37]. In West and Central Africa, only 20% of infants younger than 6 months were exclusively breastfed, and although some countries in the region such as Ghana and Nigeria have shown remarkable progress in the proportion of infants younger than 6 months who are exclusively breastfed, there is still room for improvement. Improving exclusive breastfeeding practices in West Africa would lower the risk of early introduction of solid, semi-solid or soft foods and thereby reduce the burden of infant morbidity and mortality. Breastfeeding programs should address the individual-, household- and community-level characteristics that encourage early introduction of solid, semi-solid or soft foods to the infant.
